# RNAi targeting ABCB1-like efflux transporters improves miticide efficacy in resistant *Varroa* mites

**DOI:** 10.1186/s13071-026-07461-7

**Published:** 2026-06-03

**Authors:** Vincent A. Ricigliano, Julia D. Fine, Rebecca Mueller, Laura Rivera, Eliza M. Litsey, Michelle C. Lucadello, Frank D. Rinkevich, Brian G. Rector, Sascha C. T. Nicklisch

**Affiliations:** 1 Invasive Species and Pollinator Health Research Unit, USDA-ARS, Davis, CA USA; 2 Department of Environmental Toxicology, University of California-Davis, Davis, CA USA; 3 Honey Bee Breeding Genetics and Physiology Research Unit, USDA-ARS, Baton Rouge, LA USA

**Keywords:** *Varroa destructor*, Honeybee, Amitraz resistance, RNA interference, ABC transporters

## Abstract

**Background:**

The ectoparasitic mite *Varroa destructor* is the gravest threat to managed honeybees, and its control relies on a limited number of chemical miticides. Among these, amitraz is widely used because of its strong efficacy against mites and relatively low toxicity to bees. However, increasing resistance to amitraz in *Varroa* populations threatens its long-term effectiveness. While mutations in the mite’s β2 octopamine receptor are strongly associated with amitraz resistance, additional mechanisms influencing toxicant uptake and efflux are believed to also contribute. ATP-binding cassette (ABC) transporters, including ABCB1/P-glycoproteins, are well-established mediators of xenobiotic efflux and pesticide tolerance across arthropods, making them promising targets for silencing via RNA interference (RNAi) to combat miticide resistance.

**Methods:**

We cloned a full-length *Varroa* ABCB1-like transporter (*VdABCB1*) and synthesized dsRNAs targeting its coding sequence. Adult mites were treated with dsRNA prior to amitraz exposure in laboratory bioassays. Mite survival was analyzed longitudinally, and resistance-associated β2 octopamine receptor genotypes were determined.  *Varroa* transcriptomic responses to dsRNA were assessed by RNA sequencing. Honeybee safety was evaluated in cage assays following chronic oral dsRNA exposure, including conservative tests co-administered with a known ABC transporter substrate.

**Results:**

Mites exposed to ABCB1 dsRNA showed greater amitraz-induced mortality than those treated with non-specific dsRNA. This effect was observed across multiple trials and amitraz concentrations. Transcriptomic analyses of mites revealed a significant knockdown of ABCB1-like transcripts following RNAi treatment. Chronic dietary exposure to ABCB1 dsRNA did not impact honeybee survival. Toxicity assays with ABCB1 dsRNA on its own or together with acetamiprid (ABC transporter substrate) as a high-risk interaction control showed minimal adverse effects on bees.

**Conclusions:**

RNAi suppression of *Varroa* ABCB1-like transporters increased amitraz mortality in resistant mites, identifying transporter-mediated efflux as a modifiable component of amitraz resistance. These findings demonstrate that targeted disruption of detoxification pathways can enhance miticide efficacy while minimizing off-target effects in bees. RNAi-based synergists therefore represent a selective resistance management strategy that could extend the effective lifespan of amitraz and other miticides relied on by the beekeeping industry.

**Graphical abstract:**

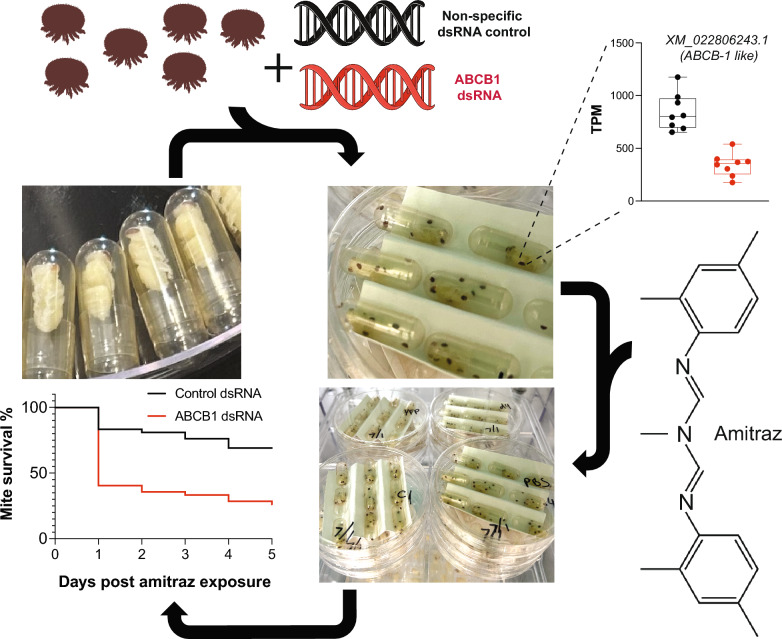

**Supplementary Information:**

The online version contains supplementary material available at 10.1186/s13071-026-07461-7.

## Background

Managed honeybees (*APis mellifera,* L.) provide essential pollination services to global agriculture, yet colony losses remain persistently high across regions and management systems [1]. Among the multiple stressors affecting honeybee health, the ectoparasitic mite *Varroa destructor* is widely recognized as the primary biotic driver of colony collapse, both through direct parasitism and as an efficient vector of pathogenic viruses [2–4]. Effective and sustainable *Varroa* control therefore remains central to efforts aimed at stabilizing managed honeybee populations [5, 6].

Chemical miticides remain the primary tool for *Varroa* management, but the limited number of registered active ingredients and the rapid evolution of resistance in *Varroa* threaten the durability of these treatments [7–9]. Amitraz, a formamidine acaricide commonly deployed via strip- or gel-based formulations, has historically provided effective mite control with relatively low toxicity to honeybees [10]. However, increasing reports of treatment failure and the spread of resistance-associated mutations in *Varroa* populations have raised concern that amitraz's efficacy may continue to erode in the absence of new resistance management strategies [9, 11, 12]. Importantly, both synthetic and organic acaricides can have unintended sublethal effects on honeybees and may accumulate in hive products, raising additional concerns for colony health and product quality [6]. Accordingly, strategies that reduce reliance on chemical treatments or enable their use at lower effective doses may provide meaningful benefits for both bee health and the sustainability of beekeeping practices.

Although target-site mutations in the *Varroa* octopamine receptor are strongly associated with amitraz resistance [11, 13–15], chemical resistance phenotypes in other organisms often reflect mechanisms that influence uptake, distribution, and detoxification [16–19]. ATP-binding cassette (ABC) transporters constitute a ubiquitous xenobiotic defense system across arthropods [20], actively exporting a wide range of compounds and thereby limiting intracellular toxicant accumulation [21]. In many arthropod species, ABCB1/P-glycoprotein transporters have been implicated in pesticide resistance, and inhibiting transporter activity can chemosensitize them by increasing cellular retention of substrate compounds [22–24].

ABC transporter activity was recently shown to contribute to amitraz tolerance in *V. destructor* [25]. Pharmacological inhibition of transporter activity enhanced amitraz-induced mortality in mites, including those with genotypes associated with amitraz resistance [25]. These findings support the hypothesis that amitraz functions as a substrate for *Varroa* ABC-mediated efflux. Thus, transporter-mediated efflux can be considered a modifiable component of amitraz resistance, and disrupting this mechanism could restore miticide efficacy. However, the use of broad-spectrum pharmacological inhibitors such as verapamil [26] in honeybee colonies poses a fundamental challenge. ABC transporters are evolutionarily conserved and play important roles in honeybee detoxification and chemical tolerance, raising the possibility that nonselective inhibitors could inadvertently chemosensitize bees or alter colony-level responses to agrochemicals [27].

To date, no *Varroa*-specific inhibitors of ABC transporters have been identified. An alternative strategy would be to selectively suppress mite transporter function using RNA interference (RNAi), a conserved gene-silencing pathway in which double-stranded RNA (dsRNA) triggers sequence-specific degradation of target transcripts [28, 29]. RNAi has been explored as a management tool for *Varroa*, primarily by directly targeting essential mite genes to reduce survival or reproduction [30–34]. In other organisms, RNAi-based approaches have been used to enhance drug efficacy by targeting disruption of resistance-associated pathways [35–37]. As such, RNAi may offer a means to selectively disrupt mite detoxification mechanisms, such as ABC transporter-mediated efflux.

Here, a dsRNA synergist targeting *Varroa* ABCB1 to increase mite sensitivity to amitraz was evaluated. This ABC transporter was selected because of its ubiquitous role in arthropod pesticide resistance. A full-length *V. destructor* ABCB1 coding sequence was cloned, multiple dsRNAs were screened, and RNAi-amitraz bioassays were conducted. To evaluate broader effects of ABCB1 perturbation, transcriptional responses were assessed by RNA sequencing (RNA-seq) on pools of mites exposed to ABCB1-targeting dsRNA. Finally, honeybee toxicity assays were performed to test potential adverse effects of dsRNA exposure. Collectively, this work establishes an RNAi-enabled chemosensitization framework that supports resistance management and extends the utility of amitraz for *Varroa* control.

## Methods

### Cloning of *V. destructor* ABCB1 (*VdABCB1*)

The complementary DNA (cDNA) sequence of a *V. destructor* ABCB-family transporter (*VdABCB1*) was identified by using the *Homo saPiens* MDR1/ABCB1 protein as a query in tBLASTn searches against the *V. destructor* transcriptome and genome assemblies. The predicted *VdABCB1* amino acid sequence showed clear similarity to well-characterized ABCB1/P-glycoproteins from human, fly, and nematode (Additional file 1: Fig. S1). The full-length *VdABCB1* coding sequence was amplified from adult mite cDNA using primers designed against the NCBI reference transcript XM_022806243.1. Total RNA was extracted from pooled mites using the RNeasy Mini Kit (Qiagen), and first-strand cDNA was synthesized with SuperScript IV Reverse Transcriptase (Thermo Fisher). PCR amplification was performed using Q5 High-Fidelity DNA Polymerase (New England Biolabs), and the resulting amplicon was gel-purified and ligated into the pCR-Blunt II-TOPO vector (Invitrogen) according to the manufacturer’s instructions. Positive clones were verified by whole-plasmid nanopore sequencing.

### dsRNA design and synthesis

To identify effective RNAi targets within the *Varroa* ABCB1 gene, three non-overlapping dsRNAs (473–480 bp) were designed corresponding to distinct coding regions (Additional file 1: Fig. S2). Since RNAi-based approaches for *Varroa* control must avoid unintended effects on honeybee health, we evaluated candidate dsRNA sequences for potential off-target interactions with the *APis mellifera* genome. Sequence similarity searches were used to ensure that the dsRNAs had minimal complementarity to bee transcripts. A dsRNA targeting yellow fluorescent protein (YFP-dsRNA; 452 bp) was used as a non-specific dsRNA control and was derived from a previously published study from our laboratory [38]. All dsRNA sequences used in this work are provided in Additional file 1: Table S1.

dsRNAs were synthesized by in vitro transcription (IVT) using T7 RNA polymerase and a HiScribe® T7 High Yield RNA Synthesis Kit (New England Biolabs) following the manufacturer’s instructions. Briefly, templates for transcription were generated from plasmid templates by PCR amplification of T7 promoter-flanked DNA fragments using gene-specific primers (listed in Additional file 1: Table S2). PCR products were verified by agarose gel electrophoresis, purified, and used directly as templates for T7-driven RNA synthesis, where sense and antisense strands were transcribed from DNA template in the same reaction [39]. Following IVT, products were purified using a Monarch® Spin RNA Cleanup Kit (New England Biolabs) and eluted in RNase-free water. RNAs were heat-denatured at 95 °C for 2 min and allowed to cool slowly to room temperature to facilitate formation of dsRNA and then quantified by spectrophotometry.

### dsRNA treatment and amitraz exposure assay

Adult *Varroa* mites were collected from heavily infested honeybee colonies at an isolated apiary using powdered sugar to dislodge mites from adult worker bees. Mites were taken directly back to the USDA-ARS Pollinator Health Laboratory and placed onto damp paper towels to gently clean them. dsRNAs targeting *VdABCB1* (ABCB1-dsRNA) or a non-specific control (YFP-dsRNA) were synthesized as described above and diluted to 2.5 µg/µl in phosphate buffered saline (PBS). Mites were randomly assigned to treatment groups and immersed in 200 µl dsRNA solutions (or PBS alone for initial control comparisons) at 4 °C for 16 h [31], and then groups of six mites were placed onto pink-eyed pupae obtained from a healthy, uninfested colony. Ten pupae per treatment (60 total mites per treatment) were loaded into size 00 gelatin capsules and placed in a desiccator housed inside an incubator set to 27 °C. Relative humidity was maintained at ~ 70% inside the desiccator using a saturated aqueous solution of NaCl in a tray placed on the bottom.

After 48 h of parasitism, surviving mites were carefully transferred to new pink-eyed pupae and gelatin capsules for amitraz challenge. Capsules were prepared by applying amitraz dissolved in acetone to the inner capsule surface to achieve the desired final concentrations of amitraz (µg/capsule). Twenty microliters of amitraz/acetone solution was dispensed into each capsule to achieve concentrations of 0.1, 0.5, 1.0, and 2.0 µg per capsule. Using range-finding experiments, these doses were selected to be above a no-observable-effects level but not toxic enough to kill the entire test population when administered using this method. Capsules were inverted 10 times, and the acetone was allowed to fully evaporate under a fume hood overnight, resulting in capsules coated semi-uniformly with amitraz and no residual acetone. Control capsules were treated with acetone only. Groups of six dsRNA-treated mites were placed onto a pupa inside an amitraz-treated capsule (*n* = 8–10 groups per treatment) and incubated at 27 °C and ~ 70% RH. Preliminary tests indicated no significant differences in survival between PBS-soaked mites and those treated with YFP dsRNA, indicating that exposure to non-specific dsRNA did not adversely affect mite viability (Supplementary Data 1: Figure S3). Based on these results, subsequent experiments included YFP-dsRNA as the sole control.

### Mite RNA isolation

Total RNA was extracted from pools of eight adult mites per treatment group (*n* = 8 YFP dsRNA-treated and *n* = 8 ABCB1 dsRNA-treated), with biological replicates processed independently. Mites were collected into bead-beating tubes containing Lysing Matrix E (1.4-mm ceramic spheres, 0.1 mm-silica spheres, and one 4-mm glass bead, MP Biomedicals) and homogenized in 350 µl of lysis buffer in a bead mill homogenizer. Lysates were clarified by centrifugation, and the supernatant was used for RNA purification with the Monarch® Total RNA Miniprep Kit (New England Biolabs) following the manufacturer’s instructions, including on-column DNase treatment. RNA was eluted in 40 µl of nuclease-free water, and RNA yield and purity (A260/280 and A260/230) were assessed by spectroscopy.

### Genome-wide transcriptome sequencing in mites

Library preparation and RNA sequencing were performed by the QB3-Berkeley Genomics core laboratories. Total RNA quality was assessed on an Agilent 2100 Bioanalyzer. We then enriched for mRNA using NEB oligo-dT beads (E7490L) and assessed the mRNA quality on an Agilent 4200 TapeStation. Libraries were prepared using the KAPA RNA Hyper Prep kit (Roche KK8581). Truncated universal stub adapters were ligated to cDNA fragments, which were then extended via 10 cycles of PCR using unique dual indexing primers into full-length Illumina adapters. Library quality was checked on an AATI (now Agilent) Fragment Analyzer. Library molarity was measured via quantitative PCR with the KAPA Library Quantification Kit (Roche KK4824) on a BioRad CFX Connect thermal cycler. Libraries were then pooled by molarity and sequenced on an Illumina NovaSeq X, 25B flowcell for 2 × 150 cycles, targeting at least 25 M reads per sample. Fastq files were generated and demultiplexed using Illumina BCL Convert v4 and default settings.

Raw sequences were quality filtered to trim low-quality ends and remove adapters using fastp [40]. To determine patterns of gene regulation, we mapped quality-filtered reads with salmon (v. 1.10.0) using the recommended decoy-aware indexing of the transcriptome reference [41] and ran a second read-based analysis using STAR (v. 2.7.11b), a splice-aware mapper with on-the-fly quantification of gene abundance [42] followed by RSEM (v. 1.3.3; [43]) to estimate isoform expression levels. Before the analysis, we standardized across samples using the transcripts per million (TPM) metric. Expression analyses were conducted with the edgeR (v4; [44]) package within the R (v. 4.5.2) statistical platform. We identified differentially expressed isoforms with the exact test [45] with a false discovery rate (FDR) of < 0.05 and alpha of 0.05. We visualized the larger patterns in transcripts between experimental groups using principal component analysis (PCA) with the plotMDS function in the limma package [46]. Datasets have been deposited at NCBI under BioProject ID PRJNA1429362.

### Amitraz resistance genotyping

Genomic DNA was isolated from individual *Varroa* mites using previously reported methods [25]. Individual *Varroa* mites were placed in 2-ml tubes containing 60 ul nuclease-free water and 2.8-mm ceramic beads. Samples were processed on a Bead Ruptor Elite homogenizer (OMNI-International) for two cycles at 4 m/s for 5 s with a 5-s pause between cycles. Following homogenization, lysates were centrifuged at 1000 × g for 2 min, and the resulting supernatant was used as template in a TaqMan assay to detect the Y215H mutation in the β2 octopamine receptor associated with amitraz resistance. Each plate contained three separate wells with control plasmid templates for the homozygous susceptible (SS), heterozygous (RS), and homozygous resistant (RR) genotypes to ensure accurate genotype results.

### Honeybee toxicity assays

The potential effects of ABCB1 dsRNA on adult honeybee survival were evaluated using laboratory cage assays. Newly eclosed worker bees were obtained from six colonies headed by queens of different genetic backgrounds (Italian and Pol-line stocks). Groups of 25 workers from the same source colony were placed into queen-monitoring cages, with three replicate cages per treatment, each derived from a different colony. Cages were maintained at 34.5 °C and 50–60% relative humidity and provided ad libitum access to water, pollen diet, and sucrose solution.

Following a 48-h acclimation period, bees were provided sucrose solution (50% w/w) containing either ABCB1 dsRNA or a non-specific YFP dsRNA control for 4 consecutive days. dsRNA was administered chronically in sucrose solution (66.7 µg ml⁻^1^) for 4 days, corresponding to an estimated minimum intake of ~ 1 µg dsRNA per bee per day based on pilot feeding trials. An additional control group received sucrose solution containing an equivalent volume of phosphate-buffered saline (PBS). After the treatment period, all cages were returned to untreated sucrose solution for the remainder of the experiment. Bee survival was monitored daily for up to 40 days.

A second assay was conducted to evaluate the effects of ABCB1 dsRNA co-administered with a known ABC transporter substrate and to compare this combination with a pharmacological ABC transporter inhibitor plus substrate. Newly eclosed worker bees were collected and maintained as described above, with groups of 25 bees per cage and three replicate cages per treatment, each originating from a different source colony. Treatment groups included ABCB1 dsRNA, YFP dsRNA, the pharmacological ABC transporter inhibitor verapamil, the neonicotinoid acetamiprid, and combinations of dsRNA or verapamil with acetamiprid, as well as an untreated sucrose control. Following a 48-h acclimation period, bees were first exposed to dsRNA treatments delivered in sucrose solution for 4 days, as described above. Subsequently, following preliminary optimizations, verapamil (1 mM) and/or acetamiprid (0.3 mM) was administered in sucrose solution for an additional 4 days, after which all cages were returned to untreated sucrose solution. Cages in the control, ABCB1 dsRNA, and YFP dsRNA treatment groups received a sucrose solution containing 0.5% acetone to account for its use as a solvent in the chemical treatment groups. Supplementary Data 1: Table S3 describes the dilution schemes used to achieve the treatment concentrations for each group. This design provided a conservative assessment of potential interactions between dsRNA exposure and ABC transporter substrates at concentrations those expected in the environment. Bee mortality was recorded daily for 15 days.

### Statistical analyses

Treatment-associated differences in survivorship were analyzed using Kaplan-Meier survival analysis followed by log-rank tests with Benjamini-Hochberg correction for multiple comparisons. Differences between treatment groups were evaluated using pairwise log-rank tests.

## Results

### Cloning and sequence analysis of *VdABCB1*

To validate the transporter subfamily classification and its potential functional roles, we performed BLAST searches against the nucleotide and translated protein databases using the full-length *VdABCB1* coding sequence (4251 bp encoding 1417 amino acids). The initial nucleotide-based BLAST searches returned ABCB1-like transcript matches only within *Varroa* species (*V. destructor* and *V. jacobsoni*), indicating that genomic resources for *Varroa* mites are limited and that these mites are phylogenetically distant from other well-characterized arthropod model systems. A protein-level BLAST and sequence alignment showed increased conservation with approximately 30% sequence similarity to other functionally characterized ABCB1 transporters across diverse taxa (Additional file 1: Fig. S1). Notably, the aligned VdABCB1 transporter sequence was longer than that of any other organism—on average 10% or 140 amino acids—which in part can account for the low sequence conservation with the other ABCB1 transporters. A conserved domain analysis using NCBI's CDSearch and InterPro of VdABCB1 revealed a Type 1 exporter architecture and classified the transporter as a member of the ABCB subfamily (also known as the MDR/TAP subfamily). Both a consensus prediction of the membrane topology and a homology model of *VdABCB1* revealed 12 distinct transmembrane domains with cytoplasmic *N*- and C-terminal extensions, which are characteristic of ABCB1 orthologs (Additional file 1: Fig. S4). As such, we describe the cloned *VdABCB1* as an ABCB1-like transporter that belongs to the P-glycoprotein/MDR subfamily.

### *VdABCB1 *RNAi enhances amitraz-induced mortality in resistant *Varroa* mites

Three non-overlapping dsRNAs targeting *VdABCB1* were evaluated to determine which provided the best RNAi efficacy. Preliminary tests indicated that two of the three dsRNAs decreased mite survival relative to non-specific YFP dsRNA and PBS control treatments following amitraz exposure (Additional file 1: Fig. S3). The construct with the highest mite mortality (ABCB1 dsRNA 2) was confirmed in a separate trial (Additional file 1: Fig. S5) and chosen for all following experiments as the ABCB1 dsRNA treatment.

**Fig. 1:**
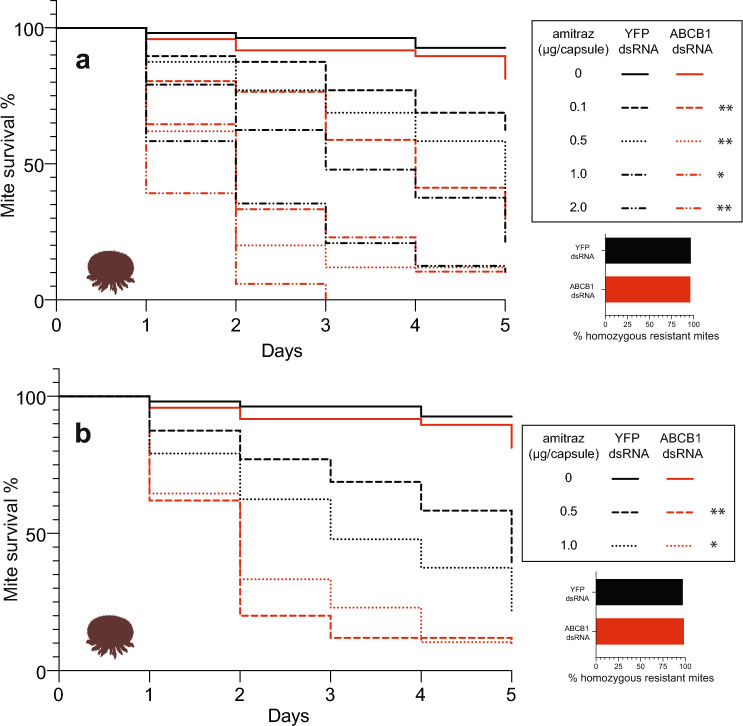
*ABCB1* dsRNA treatment potentiates amitraz-induced mortality in resistant *Varroa* mites. Adult *Varroa* mites were administered ABCB1-targeting dsRNA or a non-specific YFP dsRNA control and subsequently transferred to bee pupae housed in gelatin capsules impregnated with amitraz. Mite survival was monitored daily for 5 days following amitraz exposure. **A** Range-finding experiment assessing the combined effects of dsRNA treatment and amitraz dose on *Varroa* survival. Mite survival declined as amitraz concentrations increased. At each amitraz dose (0.1, 0.5, 1.0, and 2.0 µg per capsule), ABCB1 dsRNA-exposed mites exhibited significantly greater mortality than YFP dsRNA-treated controls (Kaplan-Meier survival analysis, λ^2^ = 282, df = 9, *N* = 494, *P* < 0.0001; see Additional file 1: Table S4 for a full list of Log-Rank comparisons). **B** Confirmation of increased amitraz sensitivity following ABCB1 dsRNA exposure. At the amitraz doses tested (0.5 and 1.0 µg per capsule), mites treated with ABCB1 dsRNA exhibited significantly higher mortality than YFP dsRNA-treated controls (Kaplan-Meier survival analysis, λ^2^ = 161, df = 5, *N* = 296, *P* < 0.0001; Additional file 1: Table S5 for a full list of Log-Rank comparisons). Asterisks indicate statistical significance: *P < 0.05; ** *P* < 0.01. In both experiments and across all treatment groups, 95% of mites were homozygous for the β2 octopamine receptor mutation associated with amitraz resistance (Y215H), indicating that ABCB1 dsRNA-mediated chemosensitization occurred in a strongly amitraz-resistant genetic background

A range-finding experiment was conducted to test the effects of dsRNA treatments combined with different doses of amitraz. Mite survival decreased in a dose-dependent manner with increasing amitraz concentrations (0, 0.1, 0.5, 1.0, 2.0 µg/capsule). At each amitraz dose, mites pre-treated with *Vd*ABCB1 dsRNA exhibited significantly higher mortality than those treated with the same dose following non-specific YFP dsRNA exposure (KM, *λ*^2^ = 282, df = 9, *N* = 494, *P* < 0.0001; Fig. 1a). To contextualize bioassay outcomes, mites from each treatment group were genotyped for the β2 octopamine receptor mutation associated with amitraz resistance. Genotyping indicated that 97% of mites in the YFP-dsRNA group and 96% of mites in the ABCB1-dsRNA group were homozygous for the β2 octopamine receptor mutation (Fig. 1a).

A second experiment was performed to confirm the impact of dsRNA treatment on amitraz sensitivity. At each of the tested amitraz doses (0, 0.5, 1.0 µg/capsule), ABCB1-dsRNA pre-exposure significantly increased mite mortality relative to nonspecific YFP dsRNA exposure (KM, *λ*^2^ = 161, df = 5, *N* = 296, *P* < 0.0001; Fig. 1b). Mites used in this assay were similarly enriched for the resistance-associated octopamine receptor mutation, with 97% (YFP-dsRNA) and 99% (*Vd*ABCB1-dsRNA) homozygous individuals in the treatment groups (Fig. 1b).

### Mite transcriptome sequencing

**Fig. 2:**
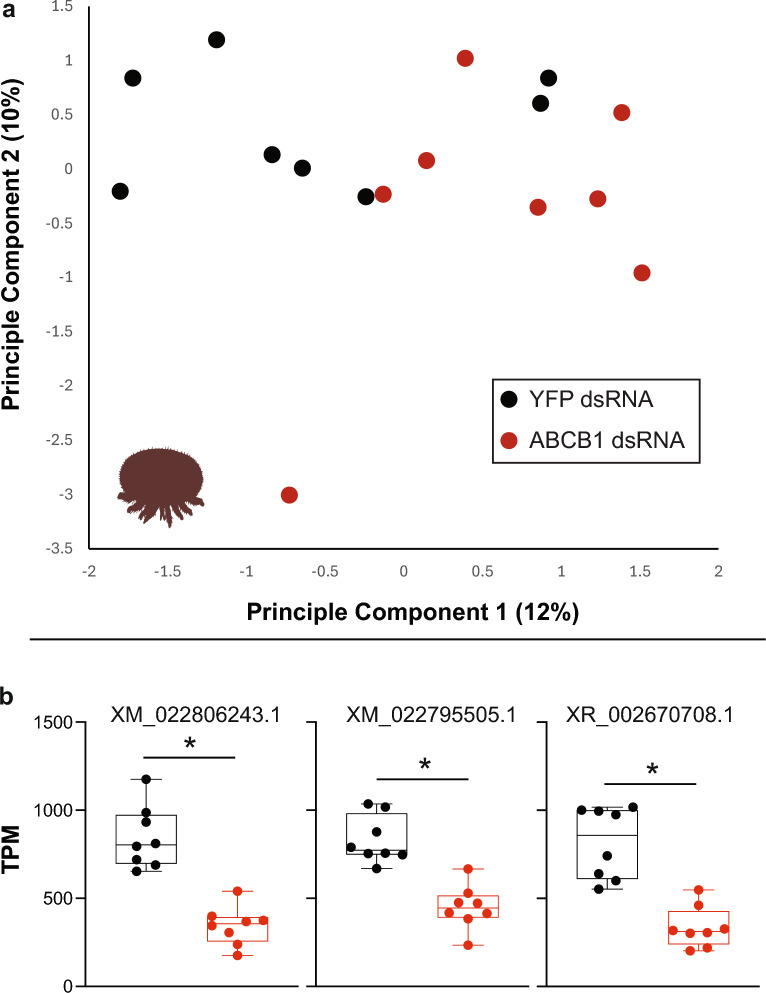
Transcriptomic responses of *Varroa* mites to ABCB1 dsRNA treatment. **a** Principal component analysis (PCA) of RNA-seq libraries from mites treated with control YFP dsRNA and ABCB1-targeting dsRNA. Each point represents an individual biological replicate. Limited separation between treatment groups is consistent with the targeted nature of RNAi, which was expected to affect a small subset of transcripts. **b** Differentially expressed transcripts identified by edgeR analysis (FDR-adjusted; * *P* < 0.05). Three transcripts were significantly downregulated in ABCB1 dsRNA-treated mites: the target ABCB1-like transporter (XM_022806243.1) and two non-target transcripts annotated as a zinc finger protein 91-like gene (XM_022795505.1) and a chondroitin sulfate synthase 3-like gene (XR_002670708.1)

To assess the global transcriptional responses to ABCB1-targeting dsRNA, we performed RNA sequencing on mites treated with ABCB1 dsRNA or control YFP dsRNA (*n* = 8 biological replicates each). Principal component analysis (PCA) revealed limited global separation between ABCB1 dsRNA-treated mites and the non-specific dsRNA control, with PC1 explaining 12% of the total variance. This pattern is consistent with the targeted mechanism of RNAi, which was expected to influence a relatively small subset of transcripts rather than broadly reshaping the transcriptome (Fig. 2a). Differential expression analysis identified three significantly downregulated transcripts in ABCB1 dsRNA-treated mites (FDR-adjusted *P* < 0.05; Fig. 2b, Additional file 2). As expected, the target ABCB1-like transporter was significantly downregulated, confirming effective RNAi-mediated knockdown. In addition to the target gene, two non-target transcripts were also significantly downregulated: XM_022795505.1, a predicted zinc finger protein 91-like gene, and XR_002670708.1, a predicted chondroitin sulfate synthase 3-like gene. No other transcripts met significance thresholds under the applied criteria, indicating a limited and specific transcriptional response to the ABCB1 dsRNA treatment.

### Honeybee toxicity assays

**Fig. 3:**
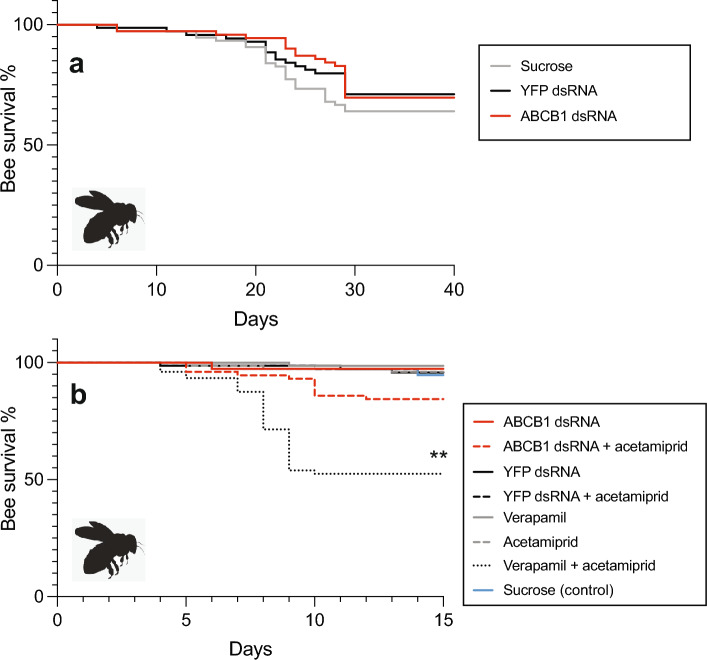
Effects of ABCB1 dsRNA on adult honeybee survival in laboratory toxicity assays. **A** Kaplan-Meier survival analysis of adult worker bees following chronic dietary exposure to ABCB1 dsRNA, a non-specific YFP dsRNA control, or sucrose-only control. Oral administration of dsRNA in sugar syrup did not significantly affect bee survival after 40 days (Kaplan-Meier survival analysis, λ^2^ = 1.5, df = 2, *N* = 225, *P* = 0.5). **B** Kaplan-Meier survival analysis of adult worker bees fed ABCB1 dsRNA, YFP dsRNA, the ABC transporter inhibitor verapamil, the neonicotinoid acetamiprid, or combinations thereof. The assay was designed to determine whether ABCB1 dsRNA exposure alters honeybee tolerance to xenobiotics by monitoring survival in the presence of a known ABC transporter substrate (acetamiprid), with comparison to a pharmacological ABC transporter inhibitor (verapamil). Treatment had a significant impact on the longevity of bees 15 days post-treatment (KM, λ^2^ = 146, df = 7, *N* = 600, *P* ≤ 0.0001). Bees in the ABCB1 dsRNA + acetamiprid group showed no difference in longevity compared with bees in the sucrose control group (*P* = 0.09). Bees in the verapamil + acetamiprid group had significantly reduced longevity compared with the sucrose control group (**P < 0.0001; see Additional file 1: Table S6 for a full list of log-rank comparisons)

To assess whether RNAi directed against *VdABCB1* produced unintended effects on honeybee survival, an oral dsRNA exposure assay was performed, which indicated that the dsRNA did not negatively impact bees when fed in sugar syrup (KM, *λ*^2^ = 1.5, df = 2, *N* = 225, *P* = 0.5; Fig. 3a).

Since transporter inhibition can potentially alter honeybee tolerance to xenobiotics, a second toxicity assay was conducted to assess potential adverse effects of dietary exposure to *VdABCB1* dsRNA, alone or in combination with a known ABC transporter substrate, the neonicotinoid acetamiprid. A pharmacological inhibitor (verapamil) was used as a positive control for ABC transporter inhibition. In this test, treatment significantly affected the survival of adult honeybees after 15 days (KM, *λ*^2^ = 146, df = 7, *N* = 600, *P* < 0.0001, Fig. 3b). Post hoc testing revealed that bees in the ABCB1 dsRNA + acetamiprid group showed no difference in longevity compared with bees in the sucrose control group (*P* = 0.09). Bees in the verapamil + acetamiprid group had significantly reduced longevity compared with the sucrose control group (*P* < 0.0001).

## Discussion

Managing *Varroa* mites effectively is crucial to the future of beekeeping, but the long-term effectiveness of current chemical treatments is being threatened as resistance continues to develop. RNAi-based technologies that directly target essential mite genes represent a promising emerging strategy for *Varroa* control, and recent studies have demonstrated their potential under field conditions [33]. However, these approaches may require relatively high dsRNA doses, repeated applications, and efficient delivery to achieve consistent efficacy, posing practical and economic challenges. In contrast, the approach described here is not intended to directly induce mite mortality but rather to enhance the efficacy of existing acaricides by targeting detoxification and tolerance mechanisms. This study showed that disrupting the mite ABCB1-like efflux transporter by RNAi increases amitraz sensitivity. These findings extend our previous work implicating pharmacological inhibition of ABC transporters in *Varroa* chemosensitization and establish RNAi as a targeted strategy for enhancing miticide efficacy while avoiding bee toxicity associated with broad-spectrum transporter inhibitors. Similar strategies are increasingly being adopted in agricultural pests, where disruption of detoxification or efflux pathways has been shown to enhance insecticide susceptibility [47–49] and, in some cases, improve field-level control [37].

Our results suggest that amitraz resistance in *Varroa* reflects not only target-site variation within the octopaminergic signaling pathway but also processes that influence intracellular toxicant accumulation. ABC transporters are ubiquitous components of xenobiotic detoxification across arthropods, and ABCB1/P-glycoproteins in particular have been repeatedly implicated in multidrug resistance phenotypes [22–24]. dsRNA directed against *VdABCB1* consistently increased amitraz-induced mortality, indicative of disrupted efflux. Mites used in the amitraz exposure assays were overwhelmingly homozygous for the resistance-associated β2 octopamine receptor mutation (96–99% across dsRNA treatment groups). RNAi-based chemosensitization therefore represents a novel *Varroa* management strategy that can partially restore miticide performance even in resistant mite populations. Increasing miticide efficacy at a given dose may reduce the frequency or intensity of treatments, slow resistance evolution, and extend the useful lifespan of the limited miticide arsenal available to beekeepers [50], thereby acting as a stop-gap measure until lethal RNAi becomes more economical.

Transcriptomic profiling of mites supported the specificity of the RNAi treatment. Only three transcripts were significantly downregulated, including the intended ABCB1-like target. This limited set of differentially expressed genes suggests that the dsRNA construct did not broadly disrupt mite transcription but instead produced a focused perturbation. The additional downregulated transcripts were annotated as a zinc finger protein 91-like gene and a chondroitin sulfate synthase 3-like gene. This may reflect secondary regulatory or physiological consequences of altered intracellular toxicant handling rather than widespread off-target silencing. Zinc finger proteins are commonly associated with transcriptional regulation [51, 52], suggesting that transporter disruption may influence downstream regulatory networks. Likewise, modulation of a chondroitin sulfate synthase-like transcript could indicate subtle changes in cellular or extracellular matrix processes [53, 54]. Although the functional significance of these changes remains to be determined, the overall transcriptional pattern is consistent with a targeted RNAi effect accompanied by limited downstream adjustments rather than systemic transcriptomic dysregulation.

Targeting conserved detoxification pathways in mites may inadvertently harm honeybees. ABC transporters contribute to xenobiotic tolerance in bees [55, 56], and pharmacological inhibition can increase sensitivity to certain pesticides [27]. Homology searches against the *APis mellifera* genome indicated that the *Varroa*-derived dsRNA construct lacked sufficient sequence identity to predict off-target effects in bees. Because sequence-based predictions may not fully reflect biological outcomes [57–59], we evaluated whether this predicted selectivity translated to honeybee toxicity assays. *VdABCB1* dsRNA did not significantly impact bee survival over a 40-day period. In a more conservative safety test, we used an ABC transporter inhibitor, verapamil [60], and acetamiprid, a known ABC transporter substrate [61], as a high-risk interaction control. This design allowed us to compare the effects of RNAi selectivity with the consequences of pharmacological ABC inhibition under identical exposure conditions. Verapamil exposure markedly reduced bee survival when combined with acetamiprid. In contrast, chronic dietary exposure to ABCB1 dsRNA did not significantly reduce bee survival. These results indicate that the dsRNA construct may offer greater selectivity than small-molecule inhibitors. While laboratory assays cannot capture all potential sublethal or colony-level effects, the use of conservative dosing, multiple honeybee genetic backgrounds, and a known high-risk comparator provides an initial safety benchmark. We acknowledge, however, that more subtle or cumulative effects may emerge over longer time frames or under chronic exposure conditions. Future studies should therefore extend these findings by evaluating long-term and colony-level impacts, including repeated exposure scenarios and potential effects on bee physiology, behavior, and overall colony performance. As such, further toxicological evaluations and transcriptome analyses in honeybees will provide more information about potential off-target effects.

## Conclusions

In summary, perturbation of ABCB-like transporters via RNAi can sensitize *Varroa* to amitraz and improve miticide performance in resistant populations. Minimal toxicity in conservative honeybee bioassays supports the selectivity of this approach compared with pharmacological ABC transporter inhibition. However, more extensive testing in bees at multiple levels of organizational complexity are necessary to fully understand the impacts on bee health. Future research should prioritize developing scalable dsRNA formulations and delivery systems that align with established beekeeping practices. Additionally, direct evaluation of transporter functionality and amitraz translocation is needed. Multiplex dsRNAs targeting several resistance-related pathways may also enhance the long-term efficacy of existing miticides.

## Supplementary Information

Additional file 1 (DOCX 2948 KB)

Additional file 2 (XLSX 4716 KB)

## Data Availability

RNA sequencing data are available in the NCBI Sequence Read Archive under BioProject accession no. PRJNA1429362.

## Abbreviations

ABC: ATP-binding cassette
ABCB1: ATP-binding cassette subfamily B member 1
BLAST: Basic local alignment search tool
cDNA: Complementary DNA
dsRNA: Double-stranded RNA
FDR: False discovery rate
IVT: In vitro transcription
MDR1: Multidrug resistance protein 1
PCA: Principal component analysis
qPCR: Quantitative polymerase chain reaction
RNAi: RNA interference
RNA-seq: RNA sequencing
RSEM: RNA-seq by expectation–maximization
STAR: Spliced transcripts alignment to a reference
tBLASTn: Translated BLAST search
TPM: Transcripts per million
