# Supplementary material for: RNAi targeting ABCB1-like efflux transporters improves miticide efficacy in resistant Varroa mites
Source: Parasit Vectors. 2026 Jun 3;19:280. doi: 10.1186/s13071-026-07461-7 (PMC13343628; doi:10.1186/s13071-026-07461-7)
Supplement: Supplementary file 1 — Additional file 1 (DOCX 2948 KB) [file 13071_2026_7461_MOESM1_ESM.docx]

**Figure S1.** Protein sequence alignment of *V. destructor* ABCB1 (VdABCB1) with representative ABCB1/P-glycoproteins from diverse taxa. The predicted amino acid sequence of VdABCB1 was aligned with well-characterized ABCB1 transporters from *Drosophila melanogaster*, *Caenorhabditis elegans*, *Homo sapiens*, and *Stegodyphus dumicola*. Although overall amino acid identity was modest (23–28%), the alignment reveals conserved features characteristic of ABCB-family transporters, consistent with VdABCB1 being a P-glycoprotein–like xenobiotic efflux transporter.

**Figure S2.** *V. destructor* ABCB1 dsRNA target design and template generation. **a.** Schematic representation of *V. destructor* ABCB1 cDNA. Three non-overlapping dsRNAs were designed to target distinct regions spanning the transcript coding region (red bars). **b.** Agarose gel electrophoresis of PCR products corresponding to each dsRNA target region. PCR amplicons of the expected sizes were generated using T7 promoter–flanked primers and served as DNA templates for subsequent *in vitro* dsRNA synthesis.

**Table S1.** dsRNA sequences used in this study. Following preliminary tests, VdABCB1 dsRNA 2 was used for all subsequent experiments.

| ID | Purpose | Length (bp) | | Reference | |
| --- | --- | --- | --- | --- | --- |
| YFP dsRNA | **dsRNA control (non-specific dsRNA)** | **452** | | **This study** | |
| GCTACCCCGACCACATGAAGCTGCACGACTTCTTCAAGTCCGCCATGCCCGAAGGCTACGTCCAGGAGCGCACCATCTTCTTCAAGGACGACGGCAACTACAAGACCCGCGCCGAGGTGAAGTTCGAGGGCGACACCCTGGTGAACCGCATCGAGCTGAAGGGCATCGACTTCAAGGAGGACGGCAACATCCTGGGGCACAAGCTGGAGTACAACTACAACAGCCACAACGTCTATATCATGGCCGACAAGCAGAAGAACGGCATCAAGGTGAACTTCAAGATCCGCCACAACATCGAGGACGGCAGCGTGCAGCTCGCCGACCACTACCAGCAGAACACCCCCATCGGCGACGGCCCCGTGCTGCTGCCCGACAACCACTACCTGAGCTACCAGTCCGCCCTGAGCAAAGACCCCAACGAGAAGCGCGATCACATGGTCCTGCTGGAGTTC | | | | | |
| VdABCB1 dsRNA 1 | **VdABCB1 target 1** | **477** | | **This study** | |
| CATTGAAGACGGGCTGGGAAGCAAGCTTGGTCTGTTTGTTTCGAACTCCTGTACATTAATTGTCTGCACCCTTACCGCATTCGCCACGAATTGGGCCCTTTTCCTCCTTATGCTCAGTCCGCTACCGATATTTTTATTGCTGGTGGTGTTTATCGGCCAGCGTGTTTCCTTGCATCAACGTAAGGAGGGCGATCAGCTTCAGATTGCGGCATTCACCGCTAGCGAAGCGATCCACAACATTCGAGCTGTGGTTGCCTTCGGCGCTGAAGATAAGGAAGTTCTCAAATTTAGCCGTCGTTTAGATGGCAGTCAACGCCATGCGTACCGCAAGCTGGTGTGGCTGAGTGCATCATCATCCCTCATCTGGTTCTTTTTGTACGCATGCTATGCCTTAGCATTTTGGTACGGTCTAAGCAATCAGCTGCTATCTGATCGAGAAAGATCGATGCTCCCATCACACGTTCTCTGCCCCATCTT | | | | | |
| VdABCB1 dsRNA 2 | **VdABCB1 target 2** | **473** | | **This study** | |
| AGCTCGAAACGCCTCGATCTTAGTTCTTGAAAATCCCGGACATAATCTTCGGGGAGATGCTGCCGCAATGGTAATACATGCCATGGAAAATGCAATGGAGGGGCGAACATCGATTCTGATCTCGAATAAAGCCTTCAAGCTGAGAGACCACGAGAGAATTATTGTTCTTGAACAAGGCAAGGTGGTTGAAGAAGGAAGCTACGCAGAGCTTGTAGCTAATAAAGGAGTTTTCGAACAGCATCTCAAAAAGGAATTACACGAGATGAAGGAGTTGGGCCTTTCGCCACTTGGTGAGGTACTTCCTCCGGCAGGTAGCATTCTCGACCGCCAGGTCTCATCGTTACTTGAACAAGATGCTTCCACTGAACATAATGAAGGATTCAATCAAGAAGCTCACCTTAAAGCAAGCTATAATCATCGTGAGCTTATCTCGCTGCTGAGGGAACATATACCCATGGTATCAGTCGGGTGTA | | | | | |
| VdABCB1 dsRNA 3 | **VdABCB1 target 3** | | **480** | | **This study** |
| TACGGAGCCTGGATGTCTCATGGGTCAGACAGCAAATGGGCATAGTAAATCTGGAGCCGCACTTTTTTCTCGACACGATCTGGGCTAACGTTGCTTATGGCCAGAGTAGAAGCCATGTCACGAATCAAATGGTAGTCACGGCCTGTCAACGCAGTCGGGTCCACGATTTCATTATTAAGCTTCCCCAAGGCTATCAAACTCTATTGGACTCATCCGGAACTCAAGGGCAGGCTATTGGCCCCACACAAAAGGTTCTACTATCTATTGCGAGAGCCGTGCTTCGTGAACCGAAAATTTTGCTGGTTGATCAGTCTGGCTGGAGCGACTTGCCCGAGGATGAGATTAGGGTAATCTTCGGTGCGCTAGAAGATGTACGAAAGGACCGCACCTGTATTGTCATCTCGCCCCTCCTGATAACAGCCGAAAAATGCGATCAGGTATGGCTGCTTCAACAAGGTCGAGTCGTAGGACAGGGCAAAC | | | | | |

**Table S2.** PCR primers used for dsRNA synthesis. T7 promoter sequences are in bold.

| Primer ID | Purpose | Reference | Sequence (5’ to 3’) |
| --- | --- | --- | --- |
| YFPdsRNA F | YFP dsRNA synthesis | This study | **TAATACGACTCACTATAGGGAGA**GCTACCCCGACCACATGAAG |
| YFPdsRNA R | YFP dsRNA synthesis | This study | **TAATACGACTCACTATAGGGAGA**GAACTCCAGCAGGACCATGT |
| ABCB1dsRNA1 F | ABCB1 dsRNA 1 synthesis | This study | **TAATACGACTCACTATAGGGAGA**ATTGAAGACGGGCTGGGAAG |
| ABCB1dsRNA1 R | ABCB1 dsRNA 1 synthesis | This study | **TAATACGACTCACTATAGGGAGA**AAGATGGGGCAGAGAACGTG |
| ABCB1dsRNA2 F | ABCB1 dsRNA 2 synthesis | This study | **TAATACGACTCACTATAGGGAGA**TAGCTCGAAACGCCTCGATC |
| ABCB1dsRNA2 R | ABCB1 dsRNA 2 synthesis | This study | **TAATACGACTCACTATAGGGAGA**CACCCGACTGATACCATGGG |
| ABCB1dsRNA3 F | ABCB1 dsRNA 3 synthesis | This study | **TAATACGACTCACTATAGGGAGA**TACGGAGCCTGGATGTCTCA |
| ABCB1dsRNA3 R | ABCB1 dsRNA 3 synthesis | This study | **TAATACGACTCACTATAGGGAGA**TGTTTGCCCTGTCCTACGAC |

**Figure S3.** Preliminary screening of ABCB1-targeting dsRNAs for effects on mite mortality. Adult *Varroa* mites were treated with one of three ABCB1-targeting dsRNAs or a non-specific YFP dsRNA control. Mite mortality was assessed 48 h after being transferred to honey bee pupae housed in gelatin capsules impregnated with 0.1 μg amitraz per capsule. Based on the results, ABCB1 dsRNA 2 was chosen for all subsequent experiments in this study. Bars represent mean ± SEM. Different letters indicate statistically significant differences among treatments (one-way ANOVA with Tukey’s multiple comparisons test, α = 0.05).

**Table S3**. Concentrations of verapamil, acetamiprid, and acetone used in bee toxicity assays

| **Treatment Group** | **Concentration in diet** | **Treatment Stock** | | **Diet** | |
| --- | --- | --- | --- | --- | --- |
|  |  | **Active Ingredient (g)** | **Acetone (mL)** | **Treatment Stock (mL)** | **50% sucrose w/w (mL)** |
| Acetamiprid | 0.3 mM | 0.00835 | 0.625 | 0.25 | 49.75 |
| Verapamil | 1 mM | 0.04546 | 0.5 | 0.25 | 49.75 |
| Acetamiprid + Verapamil | 0.3 mM acetamiprid | 0.00668 | 0.25 | 0.125 | 49.75 |
|  | 1 mM verapamil | 0.04546 | 0.25 | 0.125 |  |
| Control |  |  | 1 | 0.25 | 49.75 |


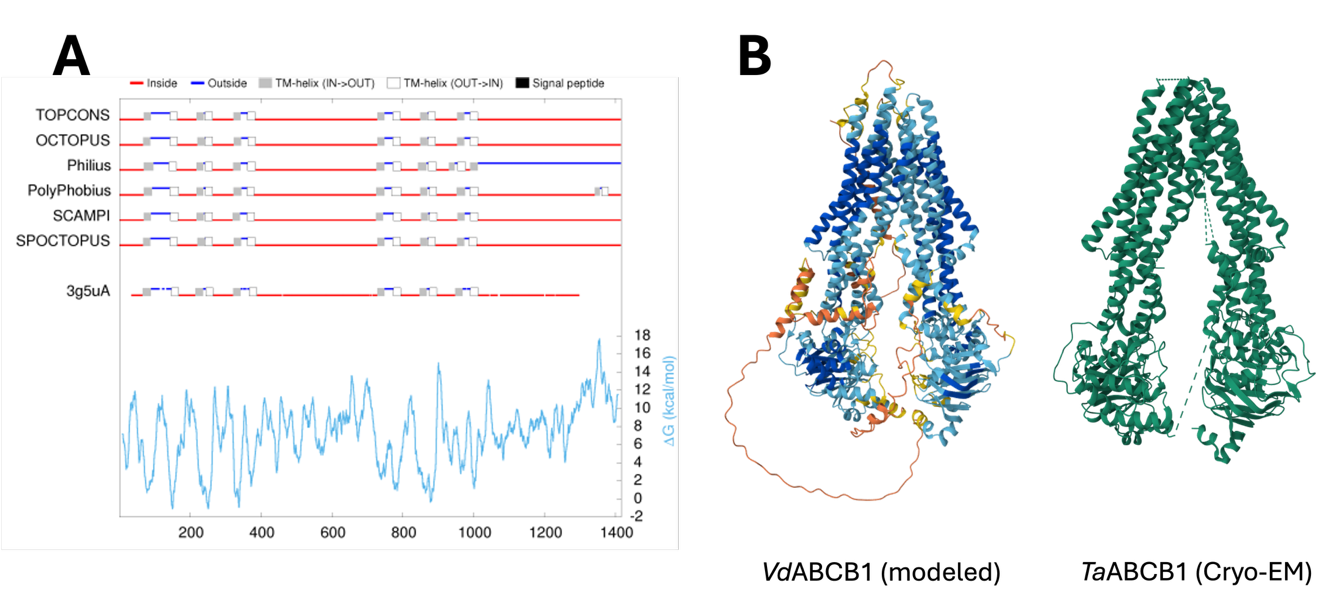


**Figure S4. Predicted Membrane Topology of *Vd*ABCB1.** (**A**) Shown are the consensus prediction and the respective reliability score across the amino acid sequence using TOPCONS (<https://topcons.net>). Inside (red) and outside (blue) orientations of the predicted membrane spanning segments relative to the membrane are displayed. The predicted 12 TM helices are highlighted and go from outside to inside (grey) or inside to outside (white) of the membrane. (**B**) Homology model with predicted structure of *VdABCB1* using AlphaFold Server (<https://alphafoldserver.com>). Shown for comparison is the experimentally derived cryo-EM structure of yellowfin tuna (*Thunnus albacares*) ABCB1 in Apo conformation (PDB ID: 9NJF).

**Figure S5**. Additional experiment showing ABCB1 dsRNA enhances amitraz sensitivity in *V*. *destructor*. Adult mites were exposed to either ABCB1-targeting dsRNA or a non-specific YFP dsRNA control and subsequently transferred to pupae housed in gelatin capsules pre-coated with amitraz (0 or 0.1 μg per capsule). Mite survival was monitored over time following amitraz exposure. Mites receiving ABCB1 dsRNA exhibited significantly reduced survival compared with YFP dsRNA–treated controls in the presence of 0.1 μg per capsule, confirming that ABCB1 dsRNA increases amitraz sensitivity.

**Table S4.** Log-Rank comparisons for Figure 1a.

| **Treatment 1** | | **Treatment 2** | |  |
| --- | --- | --- | --- | --- |
| **Amitraz Dose (µg/capsule)** | **dsRNA Treatment** | **Amitraz Dose (µg/capsule)** | **dsRNA Treatment** | **Log-Rank p-Value** |
| 0.0 | ABCB1 | 0.1 | ABCB1 | **<0.0001** |
| 0.0 | ABCB1 | 0.5 | ABCB1 | **<0.0001** |
| 0.0 | ABCB1 | 1.0 | ABCB1 | **<0.0001** |
| 0.0 | ABCB1 | 2.0 | ABCB1 | **<0.0001** |
| 0.0 | ABCB1 | 0.0 | YFP | 0.1063 |
| 0.0 | ABCB1 | 0.1 | YFP | **0.0421** |
| 0.0 | ABCB1 | 0.5 | YFP | **<0.0001** |
| 0.0 | ABCB1 | 1.0 | YFP | **<0.0001** |
| 0.0 | ABCB1 | 2.0 | YFP | **<0.0001** |
| 0.1 | ABCB1 | 0.5 | ABCB1 | **<0.0001** |
| 0.1 | ABCB1 | 1.0 | ABCB1 | **0.0003** |
| 0.1 | ABCB1 | 2.0 | ABCB1 | **<0.0001** |
| 0.1 | ABCB1 | 0.0 | YFP | **<0.0001** |
| 0.1 | ABCB1 | 0.1 | YFP | **0.002** |
| 0.1 | ABCB1 | 0.5 | YFP | 0.2308 |
| 0.1 | ABCB1 | 1.0 | YFP | 0.3086 |
| 0.1 | ABCB1 | 2.0 | YFP | **0.0003** |
| 0.5 | ABCB1 | 1.0 | ABCB1 | 0.4952 |
| 0.5 | ABCB1 | 2.0 | ABCB1 | **0.0033** |
| 0.5 | ABCB1 | 0.0 | YFP | **<0.0001** |
| 0.5 | ABCB1 | 0.1 | YFP | **<0.0001** |
| 0.5 | ABCB1 | 0.5 | YFP | **<0.0001** |
| 0.5 | ABCB1 | 1.0 | YFP | **0.0024** |
| 0.5 | ABCB1 | 2.0 | YFP | 0.641 |
| 1.0 | ABCB1 | 2.0 | ABCB1 | **0.0001** |
| 1.0 | ABCB1 | 0.0 | YFP | **<0.0001** |
| 1.0 | ABCB1 | 0.1 | YFP | **<0.0001** |
| 1.0 | ABCB1 | 0.5 | YFP | **<0.0001** |
| 1.0 | ABCB1 | 1.0 | YFP | **0.0102** |
| 1.0 | ABCB1 | 2.0 | YFP | 0.8523 |
| 0.0 | YFP | 0.1 | YFP | **0.0003** |
| 0.0 | YFP | 0.5 | YFP | **<0.0001** |
| 0.0 | YFP | 1.0 | YFP | **<0.0001** |
| 0.0 | YFP | 2.0 | YFP | **<0.0001** |
| 0.1 | YFP | 0.5 | YFP | **0.047** |
| 0.1 | YFP | 1.0 | YFP | **<0.0001** |
| 0.1 | YFP | 2.0 | YFP | **<0.0001** |
| 0.5 | YFP | 1.0 | YFP | **0.0295** |
| 0.5 | YFP | 2.0 | YFP | **<0.0001** |
| 1.0 | YFP | 2.0 | YFP | **0.0088** |

**Table S5.** Log-Rank comparisons for Figure 1b.

| **Treatment 1** | | **Treatment 2** | |  |
| --- | --- | --- | --- | --- |
| **Amitraz Dose (µg/capsule)** | **dsRNA Treatment** | **Amitraz Dose (µg/capsule)** | **dsRNA Treatment** | **Log-Rank p-Value** |
| 0.0 | ABCB1 | 0.5 | ABCB1 | **<0.0001** |
| 0.0 | ABCB1 | 1.0 | ABCB1 | **<0.0001** |
| 0.0 | ABCB1 | 0.0 | YFP | 0.1013 |
| 0.0 | ABCB1 | 0.5 | YFP | **<0.0001** |
| 0.0 | ABCB1 | 1.0 | YFP | **<0.0001** |
| 0.5 | ABCB1 | 1.0 | ABCB1 | 0.4732 |
| 0.5 | ABCB1 | 0.0 | YFP | **<0.0001** |
| 0.5 | ABCB1 | 0.5 | YFP | **<0.0001** |
| 0.5 | ABCB1 | 1.0 | YFP | **0.0024** |
| 1.0 | ABCB1 | 0.0 | YFP | 0.1013 |
| 1.0 | ABCB1 | 0.5 | YFP | **<0.0001** |
| 1.0 | ABCB1 | 1.0 | YFP | **<0.0001** |
| 0.0 | YFP | 0.5 | YFP | **<0.0001** |
| 1.0 | YFP | 1.0 | YFP | **<0.0001** |
| 0.5 | YFP | 1.0 | YFP | **0.028** |

**Table S6**. Log-Rank comparisons for Figure 3b.

| **Treatment 1** | **Treatment 2** | **Log-Rank p-Value** |
| --- | --- | --- |
| Control | Acetamiprid | 0.851 |
| Control | Verapamil | 0.386 |
| Control | Acetamiprid+Verapamil | **<0.0001** |
| Control | ABCB1 dsRNA | 0.670 |
| Control | ABCB1 dsRNA +Acetamiprid | 0.090 |
| Control | YFP dsRNA | 0.851 |
| Control | YFP dsRNA +Acetamiprid | 0.851 |
| Acetamiprid | Verapamil | 0.563 |
| Acetamiprid | Acetamiprid+Verapamil | **<0.0001** |
| Acetamiprid | ABCB1 dsRNA | 0.851 |
| Acetamiprid | ABCB1 dsRNA +Acetamiprid | 0.052 |
| Acetamiprid | YFP dsRNA | 0.982 |
| Acetamiprid | YFP dsRNA +Acetamiprid | 0.982 |
| Verapamil | Acetamiprid+Verapamil | **<0.0001** |
| Verapamil | ABCB1 dsRNA | 0.821 |
| Verapamil | ABCB1 dsRNA +Acetamiprid | **0.009** |
| Verapamil | YFP dsRNA | 0.549 |
| Verapamil | YFP dsRNA +Acetamiprid | 0.549 |
| Acetamiprid+Verapamil | ABCB1 dsRNA | **<0.0001** |
| Acetamiprid+Verapamil | ABCB1 dsRNA +Acetamiprid | **0.0001** |
| Acetamiprid+Verapamil | YFP dsRNA | **<0.0001** |
| Acetamiprid+Verapamil | YFP dsRNA +Acetamiprid | **<0.0001** |
| ABCB1 dsRNA | ABCB1 dsRNA +Acetamiprid | **0.030** |
| ABCB1 dsRNA | YFP dsRNA | 0.851 |
| ABCB1 dsRNA | YFP dsRNA +Acetamiprid | 0.851 |
| ABCB1 dsRNA +Acetamiprid | YFP dsRNA | 0.057 |
| ABCB1 dsRNA +Acetamiprid | YFP dsRNA +Acetamiprid | 0.057 |
| YFP dsRNA | YFP dsRNA +Acetamiprid | 1.000 |
